# Characteristics of refined lymphocyte subsets changes in people living with HIV/AIDS during antiretroviral therapy period: An observation from Wuhan, China

**DOI:** 10.3389/fimmu.2023.1089379

**Published:** 2023-02-09

**Authors:** Rui Yuan, Ling Li, Wenjia Hu, Ke Zhuang, Ejuan Zhang, Yajun Yan, Ling Feng, Xiaoping Chen, Qian Cao, Hengning Ke, Xien Gui, Rongrong Yang

**Affiliations:** ^1^ Department of Infectious Diseases, Zhongnan Hospital of Wuhan University, Wuhan, Hubei, China; ^2^ Animal Biosafety Shelter Laboratory (ABSL)‐III Laboratory at the Center for Animal Experiment, State Key Laboratory of Virology, Wuhan University, Wuhan, Hubei, China; ^3^ Medical Science Research Center, Zhongnan Hospital of Wuhan University, Wuhan, China

**Keywords:** refined lymphocyte subsets, PLWHA, antiretroviral therapy, CD28, HLA-DR

## Abstract

**Background:**

To analyze the changing characteristics of continuous monitoring of refined lymphocyte subsets in people living with HIV/AIDS (PLWHA) during ART period.

**Methods:**

Refined lymphocyte subsets was continuously monitored using flow cytometry for 173 PLWHA, who were hospitalized in Zhongnan Hospital of Wuhan University from August 17, 2021 to September 14, 2022. The effect of ART status and duration of ART on changes of refined lymphocyte subsets were compared in different groups. Then, the levels of refined lymphocyte subsets in PLWHA treated for more than 10 years were compared to those of 1086 healthy individuals.

**Results:**

In addition to conventional CD4^+^ T lymphocytes and CD4^+^/CD8^+^ ratio, gradually increasing in numbers of CD3^+^CD4^+^CD45RO cells, CD3^+^CD4^+^CD45RA cells, CD45RA^+^CD3^+^CD4^+^CD25^+^CD127^low^ and CD45RO^+^CD3^+^CD4^+^CD25^+^CD127^low^ cells were found with the increase of ART duration. The number of CD4^+^CD28^+^ cells and CD8^+^CD28^+^ cells were 174/ul and 233/ul at 6 months post-ART, which gradually increased to 616/ul and 461/ul after ART initiation more than 10 years. Moreover, in ART ≤ 6 months, 6 months-3years, 3-10 years and >10 years groups, the percentage of CD3^+^CD8^+^HLA^-^DR^+^/CD8 were 79.66%, 69.73%, 60.19% and 57.90%, respectively, and the differences between groups showed statistical significance (*F*=5.727, *P*=0.001). For those PLWHA with ART more than 10 years, the levels of CD4^+^ T lymphocytes, CD3^+^CD4^+^CD45RO cells, CD3^+^CD4^+^CD45RA cells, CD4^+^CD28^+^ cells and CD8^+^CD28^+^ cells can increase to levels similar to those of healthy control. However, for those PLWHA with ART more than 10 years, CD4^+^/CD8^+^ ratio was 0.86 ± 0.47, which was lower than that of healthy control (0.86 ± 0.47 vs 1.32 ± 0.59, *t*=3.611, *P*=0.003); absolute counts and percentage of CD3^+^CD8^+^HLA^-^DR^+^ cells were 547/ul and 57.90%, which were higher than those of healthy control(547/ul vs 135/ul, *t*=3.612, *P*=0.003; 57.90% vs 22.38%, *t*=6.959, *P<*0.001).

**Conclusion:**

Persistent ART can gradually improve the immune status of PLWHA, which is manifested in the increase of lymphocytes, function recovery of lymphocytes and reduction of aberrant activation status of the immune system. After 10 years of standardized ART, most lymphocytes could return to levels of healthy persons, although it may take longer to complete recovery for CD4^+^/CD8^+^ ratio and CD3^+^CD8^+^HLA^-^DR^+^ cells.

## Introduction

Acquired immunodeficiency syndrome (AIDS) is a chronic infectious disease caused by human immunodeficiency virus (HIV), which is one of the most destructive epidemics and the impact goes beyond public health concerns (1). It is characterized by the destruction of the function of the autoimmune system, resulting in a progressive loss of CD4+ T lymphocytes and a diverse range of immunological abnormalities, which eventually leads to various opportunistic infections and carcinomas (2). Antiretroviral therapy (ART) is the most effective clinical treatment for AIDS, which can effectively inhibit the replication of HIV and promote the recovery of immune function (3), and thereby prolonging the survival time and controlling complications (4). CD4^+^ T lymphocytes is one of the routine monitoring indicators to evaluate the efficacy during ART period. Many studies reported the characteristics and significance of changes in lymphocyte subsets in disease progression and clinical outcomes in people living with HIV/AIDS (PLWHA). Notably, most of the previous studies have focused merely on CD4^+^ T lymphocyte, whereas comprehensive interpretation and study of refined lymphocyte subtypes are rare. However, it is not only the counts of lymphocytes decrease after HIV infection, but can also cause to the defective function of immune cells and the abnormal activation of the immune system. Thus, it is particularly critical to comprehensively evaluate the changes of overall immune status in PLWHA during ART period.

A wide variety of lymphocytes are available, of which different types express antigens with specificity and perform various biological functions. Moreover, flow cytometry can be used to make a more refined classification of lymphocytes by taking advantage of the molecular differences of antigens expressed on the cell surface. In this study, refined lymphocyte subsets was continuously monitored using flow cytometry for 173 PLWHA, and the changes of lymphocytes were observed at different time during ART period. Then, the levels of refined lymphocyte subsets in PLWHA treated for more than 10 years were compared to those of 1086 healthy individuals. This study can provide clinical data for in-depth study of immune reconstitution strategies, and further guide the clinical treatment and precise monitoring of the disease.

## Materials and methods

### Study population

A total of 173 hospitalized PLWHA in Zhongnan Hospital of Wuhan University from August 17, 2021 to September 14, 2022 were selected as study subjects. Demographic and clinical information including age, gender, refined lymphocyte subsets data, duration time of ART were collected from the “HIS system of Zhongnan Hospital in Wuhan University”. There were 139 males and 34 females in this study, and their mean age was 41.3 years. HIV RNA was detectable in all PLWHA prior to ART, ranging from 1.0×10^4^ copies/ml to 1.0×10^6^ copies/ml. The inclusion criteria were: 1) confirmed HIV infection; 2) received refined lymphocyte subsets detection; 3)had a definite duration time of ART and maintained good compliance. The exclusion criteria were: 1) age< 18 years old or age>70 years old; 2) pregnant woman; 3) had immunodeficiency diseases other than HIV; 4) had carcinoma; 5) used immunosuppressive drugs;6) had COVID-19 infections. Meanwhile, 1086 healthy individuals were detected for refined lymphocyte subsets as control population.

This study was approved by the Ethics Committee of Zhongnan Hospital of Wuhan University, and all individuals signed an informed consent form.

### Study protocol

Using the time of inclusion in the study as the starting point, 173 PLWHA were divided into ART-naive and ART groups based on whether ART was initiated, whose peripheral blood was collected and tested for refined lymphocyte subsets using flow cytometry. Those PLWHA with ART were further grouped into different groups according to the duration time of ART, and the dynamic changes of refined lymphocyte subsets were analyzed and described. Then, the levels of refined lymphocyte subsets in PLWHA treated for more than ten years were compared to 1086 healthy individuals.

### Sample preparation

A vacuum collection tube with EDTA-K2 anticoagulation was used to collect 3-5 ml of peripheral venous blood from the test subject, and the tube was immediately held at both ends and mixed several times upside down vertically to prevent blood clotting. The blood sample was stored and transported at ambient temperature for testing within 24 h.

### Antibody preparation

Take two flow-through tubes, one tube with 5ul each of APC H7, Percp, V500, PE, APC, PE cy7, and BV421 to be mixed; the other tube with 5ul each of APC H7, Percp, V500, PE, APC, BV 421, and FITC at the same time to be mixed. The FACSCalibur flow cytometer and accompanying reagents from BD, USA, were performed strictly according to the instructions and within the reagent expiration date.

### Flow cytometry detection

Take 30ul and 35ul of antibody from the above two flow-cytometry tubes, respectively, then add 100ul of whole blood specimen and incubate for 15 minutes at ambient temperature (20-25 °C) against the light after thorough mixing. Add 2ml FACS hemolysin, mix thoroughly and incubate for 15 minutes at room temperature (20-25 °C), followed by centrifugation at 1300 rpm for 5 minutes, after which the supernatant is carefully discarded. Then add 2 ml of PBS, mix well and centrifuge at 1300 rpm for 5 minutes, carefully discard the supernatant. Finally, 200ul PBS was added and blended well, with 1,000 cells obtained for assay using Multiset software.

### Gating strategy for refined lymphocytes subsets

The first flow-cytometry tube was circled as follows: (1) Cell clusters were circled on scatter plot of FSC VS SSC; (2) Lymphocytes clusters were circled on scatter plot of CD45 VS SSC; (3) CD3+ cells were circled in the lymphocyte clusters shown on scatter plot of CD3 VS SSC; (4) CD3+CD4+ cells were circled in the lymphocyte clusters shown on scatter plot of CD3 VS CD4; (5) CD3+CD8+ cells were circled in the lymphocyte clusters shown on scatter plot of CD3 VS CD8; (6) CD3+DR+ cells were circled in the lymphocyte clusters shown on scatter plot of CD3 VS HLA-DR; (7) CD3+CD8+DR+ cells were circled in the lymphocyte clusters shown on scatter plot of CD8 VS HLA-DR; (8) CD3+CD4+CD28+ cells were circled in the CD4+ T lymphocyte clusters shown on scatter plot of CD4 VS CD28; (9) CD3+CD8+CD28+ cells were circled in the CD8+ T lymphocyte clusters shown on scatter plot of CD8 VS CD28. The second flow-cytometry tube was circled as follows: (1) Cell clusters were circled on scatter plot of FSC VS SSC; (2) Lymphocytes clusters were circled on scatter plot of CD45 VS SSC; (3) CD3+CD4+ cells were circled in the lymphocyte clusters shown on scatter plot of CD3 VS CD4; (4) In CD4+ T lymphocyte clusters shown in the scatter plot of CD45RA VS CD45RO, CD3+CD4+CD45RO+ cell clusters were circled and CD3+CD4+CD45RA+ cell clusters were reversely circled; (5) CD3+CD4+CD25+CD127low+ cells were circled in the CD4+ T lymphocyte clusters shown on scatter plot of CD25 VS CD127; (6) In CD3+CD4+CD25+CD127low+ cells shown in the scatter plot of CD45RA VS CD45RO, CD45RO+CD3+CD4+CD25+CD127low+ cells clusters were circled and CD45RA+CD3+CD4+CD25+CD127low+ cells clusters were reversely circled.

### Statistical analysis

Statistical analysis software SPSS 25.0 was used for data processing, with measurement data expressed as ( $\bar{\text{x}}$ ± s) and independent sample t-test for comparison of means between two groups. ANOVA was performed for comparison of means between multiple groups and Dunnett’s t-test was conducted for a two-group comparison. For data results, Graphpad Prism 8.0 Statistical analysis was used and differences were considered statistically significant at *P*<0.05.

## Results

### Effect of ART on refined lymphocyte subsets in PLWHA

Compared with ART-naive PLWHA, the number of total lymphocytes, CD3+ T lymphocyte, CD3+CD4+T lymphocyte, CD19+ B lymphocyte and the ratio of CD4+/CD8+, CD3+CD4+/CD3+, CD3+CD8+/CD3+ were significantly increased after ART, whereas the absolute counts of CD16+CD56+NK cells reduced dramatically after ART. It was found that the differences in the percentages of CD16+CD56+ NK lymphocytes (*t*=1.667, *P*=0.097) and total T lymphocytes (*t*=0.350, *P*=0.727) were not statistically different. All these results were shown in **Table 1**.

**Table 1:** Effect of ART on conventional detection of lymphocyte subsets.

Parameters	ART-naive (n=76)	ART (n=97)	*t*	*P*
Lymphocyte counts(cells/ul)	870.98 ± 978.45	1478.54 ± 713.55	4.721	0.000
CD3+ T counts(cells/ul)	679.92 ± 848.63	1084.59 ± 553.53	3.782	0.000
CD3+ T percentage(%)	73.46 ± 12.06	72.84 ± 11.09	0.350	0.727
CD3+CD4+T counts(cells/ul)	83.05 ± 124.76	336.59 ± 266.75	8.277	0.000
CD3+CD4+/CD3+(%)	7.20 ± 7.02	21.91 ± 12.65	9.657	0.000
CD3+CD8+T counts(cells/ul)	566.24 ± 744.19	685.38 ± 408.23	1.341	0.182
CD3+CD8+/CD3+(%)	61.82 ± 13.01	46.08 ± 17.66	6.742	0.000
CD4+/CD8+(%)	0.14 ± 0.16	0.62 ± 0.51	8.760	0.000
CD19+ B counts(cells/ul)	63.69 ± 73.32	179.12 ± 152.08	6.565	0.000
CD19+ B percentage (%)	8.65 ± 8.11	11.79 ± 8.30	2.494	0.014
CD16+CD56+ NK counts(cells/ul)	127.37 ± 136.39	14.51 ± 9.07	7.201	0.000
CD16+CD56+ NK percentage(%)	16.97 ± 10.31	14.51 ± 9.06	1.667	0.097

**Figure 1:**
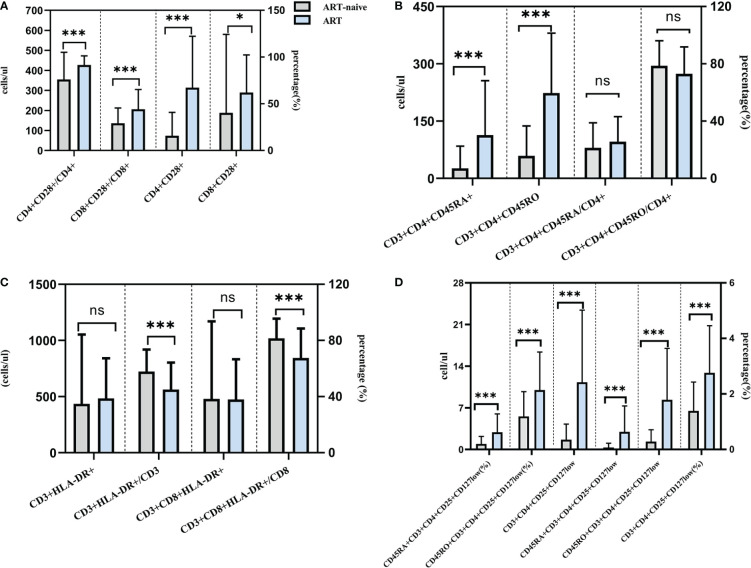
Effect of ART on lymphocyte subsets in PLWHA through refined lymphocyte subsets detection. Effect of ART treatment on CD28 expression **(A)**. Effect of ART treatment on naive and memory T cell subsets **(B)**. Effect of ART treatment on HLA-DR expression on T lymphocyte **(C)**. Effect of ART treatment on regulatory CD4^+^ T cells **(D)**. *P < 0.05, ***P < 0.001. ns, not significant.

**Figure 2:**
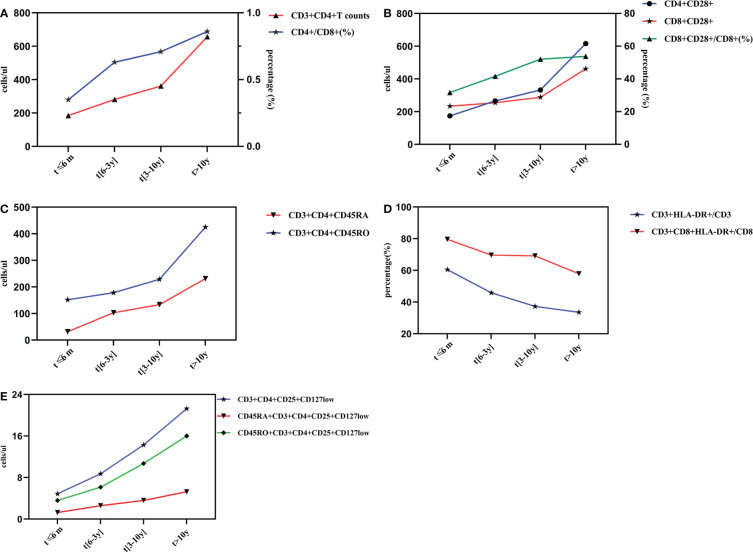
Dynamic changes of lymphocyte subsets with different time of ART implementation. Changes of Th and CD4+/CD8+ (%) after different duration time of ART **(A)**. Changes of CD28 expression after different duration of ART treatment **(B)**. Changes of naïve/memory T lymphocyte subsets after different duration time of ART **(C)**. Changes of activate T lymphocyte subsets after different duration time of ART **(D)**. Changes of regulatory T lymphocyte subsets after different duration time of ART **(E)**.

In addition to conventional lymphocytes, as shown in **Figure 1A**, the further results showed that the number of CD4+CD28+ T lymphocyte and CD8+CD28+ T lymphocyte were (314.24 ± 256.79)/ul and (289.68 ± 187.51)/ul, and the percentages were (91.54 ± 9.82)% and (44.17 ± 21.08)%, respectively, which were all significantly higher than those of ART-naive PLWHA(*P*<0.05). Besides, as shown in **Figure 2B**, the percentages of CD3+HLA-DR+ and CD3+CD8+HLA-DR+ were lower in PLWHA with ART than those ART-naive PLWHA (45.09 ± 19.19 vs 57.85 ± 15.69, 67.53 ± 20.97 vs 81.53 ± 13.97)%, whilst the number of CD3+ HLA-DR+ cells and CD3+CD8+ HLA-DR+ cells did not change significantly. In contrast, as presented in **Figure 1C**, the absolute counts of naive/memory CD4+ T lymphocytes were significantly higher than that of ART-naive PLWHA (113.26 ± 142.52 vs 26.59 ± 58.01, 223.82± 156.63 vs 59.06 ± 78.23)/ul (*P*<0.05), but the percentage of naive/memory CD4+ T lymphocytes were not significantly different between ART-initiated and ART-naive PLWHA. Accordingly, as shown in **Figure 1D**, the analysis of the regulatory T lymphocyte subsets showed that the number and percentages of regulatory lymphocyte subsets, including CD3+CD4+CD25+CD127low, CD45RA+CD3+CD4+CD25+CD127low and CD45RO+CD3+CD4+CD25+CD127low in PLWHA were significantly higher than those ART-naive PLWHA, and the difference was statistically significant (*P*<0.001).

### Effect of ART duration on CD3+CD4+T cells, CD3+CD8+T cells, and CD4/CD8 ratio

The number of CD3+CD4+ and CD3+CD8+T lymphocytes were 185.04/ul and 799.87/ul at 6 months post-ART, which gradually increased to 656.71/ul and 887.78/ul after ART initiation for more than 10 years. Although some fluctuations in the number of CD3+CD8+T cells during ART, the differences between groups showed statistical significance (*F*=13.684, *P*<0.001).The ratio of CD4+/CD8+ showed a markedly increasing trend throughout the initiation of ART, and it reached (0.86 ± 0.47)% after 10 years, and the differences in four groups were statistically significant (*F*=3.996, *P*<0.05). All these results were shown in **Table 2**, and the dynamic changes trends were shown in **Figure 2A**.

**Table 2:** Changes of CD3+CD4+T lymphocyte, CD3+CD8+T lymphocyte, and CD4+/CD8+ (%) after different duration time of ART.

Parameters	ART duration ( $\bar{\text{x}}$ ± s)				F test	
	t ≤ 6m	t [6-3y]	t [3-10y]	t > 10y	*F*	*P*
CD3+CD4+T counts(cells/ul)	185.04 ± 132.79	281.72 ± 220.85	361.46 ± 266.77	656.71 ± 263.66	3.252	0.025
CD3+CD8+T counts(cells/ul)	799.87 ± 517.99	633.27 ± 382.38	549.70 ± 222.75	887.78 ± 462.04	13.684	0.000
CD4+/CD8+(%)	0.35 ± 0.33	0.63 ± 0.62	0.71 ± 0.44	0.86 ± 0.47	3.996	0.010

### Changes of CD4+CD28+ T cells and CD8+CD28+ T cells with ART duration

As observed from **Table 3**, the number of CD4+CD28+ and CD8+CD28+ T lymphocyte gradully increased during ART period, and reached to (616.32 ± 270.99)/ul and (461.44± 237.80)/ul after ART initiation for more than 10 years, respectively. There were significantly differences between groups. Similarly, the CD8+CD28+/CD8+ ratio showed a similar trend. However, CD4+CD28+/CD4+ (%) showed a tendency of obvious fluctuations and no statistically significant differences were observed. The dynamic changes trends were shown in **Figure 2B**.

**Table 3:** Changes of CD28 molecular after different duration of ART treatment.

Parameters	ART duration ( $\bar{\text{x}}$ ± s)				F test	
	t ≤ 6m	t [6-3y]	t [3-10y]	t > 10y	*F*	*P*
CD4+CD28+(cells/ul)	174.18 ± 128.24	265.88 ± 219.31	332.04 ± 250.86	616.32 ± 270.99	12.564	0.000
CD4+CD28+/CD4+(%)	90.63 ± 12.53	92.80 ± 8.72	90.41 ± 9.64	92.92 ± 7.26	0.444	0.722
CD8+CD28+(cells/ul)	233.43 ± 136.10	254.49 ± 169.94	288.51 ± 174.24	461.44 ± 237.80	5.709	0.001
CD8+CD28+/CD8+(%)	31.69 ± 13.52	41.64 ± 17.67	52.09 ± 25.73	53.84 ± 16.14	6.206	0.001

### Influence of ART duration on naïve/memory T lymphocyte subsets

It was clear that CD3+CD4+CD45RA+ T lymphocytes and CD3+CD4+CD45RO+ T lymphocytes had the same trends of variation from the **Figure 2C**, and the differences between groups showed statistical significance (*P*<0.001). Specifically as shown in **Table 4**, CD3+CD4+CD45RA+ T cells were still at a low level of (31.84 ± 40.51)/ul at 6 months post-ART, which gradually rised to (231.39 ± 174.15)/ul after ART initiation for more than 10 years. Likewise, CD3+CD4+CD45RO+ T cells were still at rather depressed levels of (151.67 ± 116.59)/ul at the time of ART treatment for less than 6 months, then slowly increased to (229.00 ± 146.58)/ul after 3 to 10 years of treatment and rapidly reached to (425.31 ± 159.11)/ul after 10 years. However, the ratio of CD3+CD4+CD45RA/CD4+ and CD3+CD4+CD45RO/CD4+ fluctuated with no regularity. Excitingly, the differences between groups for the CD3+CD4+CD45RA/CD4+ ratio were statistically significant (*F*=4.198, *P*<0.05), whereas there was no difference between groups for the CD3+CD4+CD45RO/CD4+ ratio.

**Table 4:** Changes of naïve/memory T lymphocyte subsets after different duration of ART treatment.

Parameters	ART duration ( $\bar{\text{x}}$ ± s)				F test	
	t ≤ 6m	t [6-3y]	t [3-10y]	t > 10y	*F*	*P*
CD3+CD4+CD45RA+(cells/ul)	31.84 ± 40.51	103.26 ± 132.68	133.62 ± 150.63	231.39 ± 174.15	7.260	0.000
CD3+CD4+CD45RA/CD4+(%)	15.83 ± 16.88	27.88 ± 17.54	27.69 ± 16.08	33.70 ± 15.43	4.198	0.008
CD3+CD4+CD45RO(cells/ul)	151.67 ± 116.59	178.43 ± 108.68	229.00 ± 146.58	425.31 ± 159.11	14.513	0.000
CD3+CD4+CD45RO/CD4+(%)	80.33 ± 23.98	72.10 ± 17.54	71.42 ± 15.21	66.29 ± 15.43	1.976	0.123

### Impact of ART duration on activate T lymphocyte subsets

In contrast to the CD28 molecule, the HLA-DR molecule showed a trend of apparently different alterations. As shown in **Table 5**, in ART ≤ 6 months, 6 months -3years, 3 years-10 years and >10 years groups, the percentage of CD3+CD8+HLA-DR+/CD8 and CD3+HLA-DR+/CD3 were 79.66% vs 60.48%, 69.73% vs 45.93%, 60.19% vs 37.33% and 57.90% vs 33.58%, respectively, and the differences between groups showed statistical significance (*P*<0.001). The dynamic changes trends of CD3+CD8+HLA-DR+/CD8 and CD3+HLA-DR+/CD3 were shown in **Figure 2D**. Although the differences of CD3+HLA-DR+ and CD3+CD8+HLA-DR+ counts showed statistical significance in different groups, there were fluctuations in the changes during ART.

**Table 5:** Changes of activate T lymphocyte subsets after different duration of ART treatment.

Parameters	ART duration ( $\bar{\text{x}}$ ± s)				F test	
	t ≤ 6m	t [6-3y]	t [3-10y]	t > 10y	*F*	*P*
CD3+HLA-DR+(cells/ul)	664.71 ± 480.82	440.60 ± 287.52	340.25 ± 170.06	573.37 ± 413.84	4.55	0.005
CD3+HLA-DR+/CD3(%)	60.48 ± 14.89	45.93 ± 18.38	37.33 ± 16.99	33.58 ± 15.35	11.17	0.000
CD3+CD8+HLA-DR+(cells/ul)	636.85 ± 455.74	450.31 ± 318.85	332.47 ± 190.61	547.12 ± 426.89	3.728	0.014
CD3+CD8+HLA-DR+/CD8(%)	79.66 ± 15.85	69.73 ± 20.27	60.19 ± 21.65	57.90 ± 19.10	5.727	0.001

### Changes of regulatory T lymphocyte subsets during ART period

As presented in **Figure 2E**, it is obvious that CD3+CD4+CD25+CD127low T lymphocyte counts,CD45RA+CD3+CD4+CD25+CD127low T lymphocyte counts as well as CD45RO+CD3+CD4+CD25+CD127low T lymphocyte counts were gradually increased with the extention of ART. Specifically, in ART ≤ 6 months, 6 months-3years, 3 years-10 years and >10 years groups, the CD3+CD4+CD25+CD127low T lymphocyte counts were (4.86 ± 4.68, 8.73 ± 9.60, 14.28 ± 13.65, 21.27 ± 14.64)/ul, the CD45RA+CD3+CD4+CD25+CD127low T lymphocyte counts were (1.27 ± 1.91, 2.58 ± 3.58, 3.58 ± 5.40,5.27 ± 5.30)/ul, and the CD45RO+CD3+CD4+CD25+CD127low T lymphocyte counts were (3.59 ± 3.55,6.14 ± 6.73,10.70 ± 9.54,16.00 ± 9.92)/ul, respectively, which showed statistical differences between groups(*P*<0.05). As for the proportion of these three evaluated regulatory T lymphocyte subsets, there were no statistical significant differences between groups with different ART duration(*P*>0.05). All these results were shown in **Table 6**.

**Table 6:** Changes of regulatory T lymphocyte subsets after different duration of ART treatment.

Parameters	ART duration ( $\bar{\text{x}}$ ± s)				F test	
	t ≤ 6m	t [6-3y]	t [3-10y]	t > 10y	*F*	*P*
CD3+CD4+CD25+CD127low(cells/ul)	4.86 ± 4.68	8.73 ± 9.60	14.28 ± 13.65	21.27 ± 14.64	7.825	0.000
CD3+CD4+CD25+CD127low(%)	2.18 ± 1.41	2.62 ± 1.47	3.23 ± 2.22	3.02 ± 0.92	1.912	0.133
CD45RA+CD3+CD4+CD25+CD127low(cells/ul)	1.27 ± 1.91	2.58 ± 3.58	3.58 ± 5.40	5.27 ± 5.30	2.983	0.035
CD45RA+CD3+CD4+CD25+CD127low(%)	0.53 ± 0.87	0.60 ± 0.62	0.62 ± 0.61	0.70 ± 0.43	0.258	0.856
CD45RO+CD3+CD4+CD25+CD127low(cells/ul)	3.59 ± 3.55	6.14 ± 6.73	10.70 ± 9.54	16.00 ± 9.92	9.451	0.000
CD45RO+CD3+CD4+CD25+CD127low(%)	1.64 ± 1.07	2.01 ± 1.21	2.55 ± 1.80	2.32 ± 0.67	2.225	0.090

### Comparison of lymphocytes between PLWHA received ART more than 10 years and healthy persons

**Figure 3:**
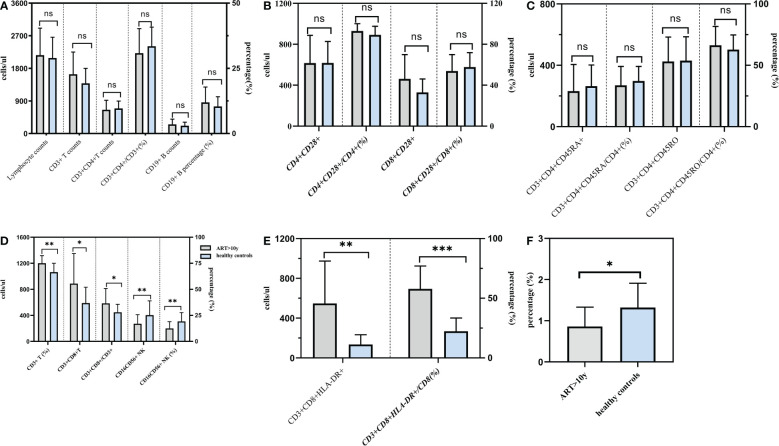
Comparison of lymphocytes between PLWHA received ART more than 10 years and healthy persons. Both T and B lymphocytes returned to normal levels after 10 years of ART **(A)**. CD28 expression returned to normal levels after 10 years of ART **(b)**. Naive/memory lymphocytes returned to normal levels after 10 years of ART **(C)**. CD8^+^ T lymphocytes and NK cells were not restored to normal levels after 10 years of ART **(D)**. HLA-DR expression were higher than healthy persons **(E)**. CD4/CD8 ratio was lower than healthy persons **(F)**. *P < 0.05, **P < 0.01.***P < 0.001. ns, not significant.

In addition to CD3+ T, CD3+CD4+T, CD19+ B lymphocyte counts and the proportion,we also find that CD4+CD28+, CD8+CD28+, CD3+CD4+CD45RA+, CD3+CD4+CD45RO+ lymphocyte counts and the proportion were significantly increased and almostly reach the level of healthy people. There was no statistical difference between the 10 years post-ART group and healthy individuals. Nevertheless, the statistical analyses in **Figure 3** showed that CD3+CD8+T lymphocyte counts and the proportion were still higher than those found in healthy individuals and the number and percentages of CD16+CD56+ NK cell were still lower than healthy people. Besides, for those PLWHA with ART more than 10 years, ratio of CD4+/CD8+ was 0.86 ± 0.47, which was lower than that of healthy control (0.86 ± 0.47 vs 1.32 ± 0.59, *t*=3.611, *P*=0.003); absolute counts and percentage of CD3+CD8+HLA-DR+ cells were 547/ul and 57.90%, which were higher than those of healthy control (547/ul vs 135/ul, *t*=3.612, *P*=0.003; 57.90% vs 22.38%, *t*=6.959, *P*<0.001). All these results were shown in **Table 7**.

**Table 7:** The comparison of lymphocytes between PLWHA received ART more than 10 years and healthy persons.

Parameters	t > 10 y (n=14)	Healthy controls (n=1068)	T test	
			*t*	*p*
Lymphocyte counts(cells/ul)	2162.92 ± 747.94	2086 ± 574	0.385	0.707
CD3+ T counts(cells/ul)	1637.00 ± 613.15	1387 ± 414	1.526	0.151
CD3+ T percentage(%)	75.00 ± 7.34	66.44 ± 8.58	4.361	0.001
CD3+CD4+T counts(cells/ul)	656.71 ± 263.66	694 ± 202	0.529	0.606
CD3+CD4+/CD3+(%)	30.81 ± 9.42	33.48 ± 7.33	1.059	0.309
CD3+CD8+T counts(cells/ul)	887.78 ± 462.04	589 ± 244	2.420	0.031
CD3+CD8+/CD3+(%)	36.56 ± 14.27	27.96 ± 7.67	2.255	0.042
CD4+/CD8+(%)	0.86 ± 0.47	1.32 ± 0.59	3.611	0.003
CD19+ B counts(cells/ul)	255.28 ± 144.21	216 ± 99	1.019	0.327
CD19+ B percentage (%)	11.96 ± 5.90	10.40 ± 3.73	0.993	0.339
CD16+CD56+ NK counts(cells/ul)	270.64 ± 140.24	403 ± 220	3.531	0.004
CD16+CD56+ NK percentage(%)	12.45 ± 6.49	19.19 ± 8.52	3.884	0.002
CD4+CD28+(cells/ul)	616.32 ± 270.99	617 ± 210	0.009	0.993
CD4+CD28+/CD4+(%)	92.92 ± 7.26	89.18 ± 8.44	1.929	0.076
CD8+CD28+(cells/ul)	461.44 ± 237.80	329 ± 132	2.084	0.057
CD8+CD28+/CD8+(%)	53.84 ± 16.14	57.71 ± 14.17	0.895	0.387
CD3+CD8+HLA-DR+(cells/ul)	547.12 ± 426.89	135 ± 98	3.612	0.003
CD3+CD8+HLA-DR+/CD8(%)	57.90 ± 19.10	22.38 ± 11.08	6.959	0.000
CD3+CD4+CD45RA+(cells/ul)	231.39 ± 174.15	264 ± 138	0.700	0.496
CD3+CD4+CD45RA/CD4+(%)	33.70 ± 15.43	37.19 ± 11.94	0.846	0.413
CD3+CD4+CD45RO(cells/ul)	425.31 ± 159.11	430 ± 156	0.110	0.914
CD3+CD4+CD45RO/CD4+(%)	66.29 ± 15.43	62.81 ± 11.94	0.846	0.413

### The correlation between HIV RNA viral load and refined lymphocytes subsets

**Figure 4:**
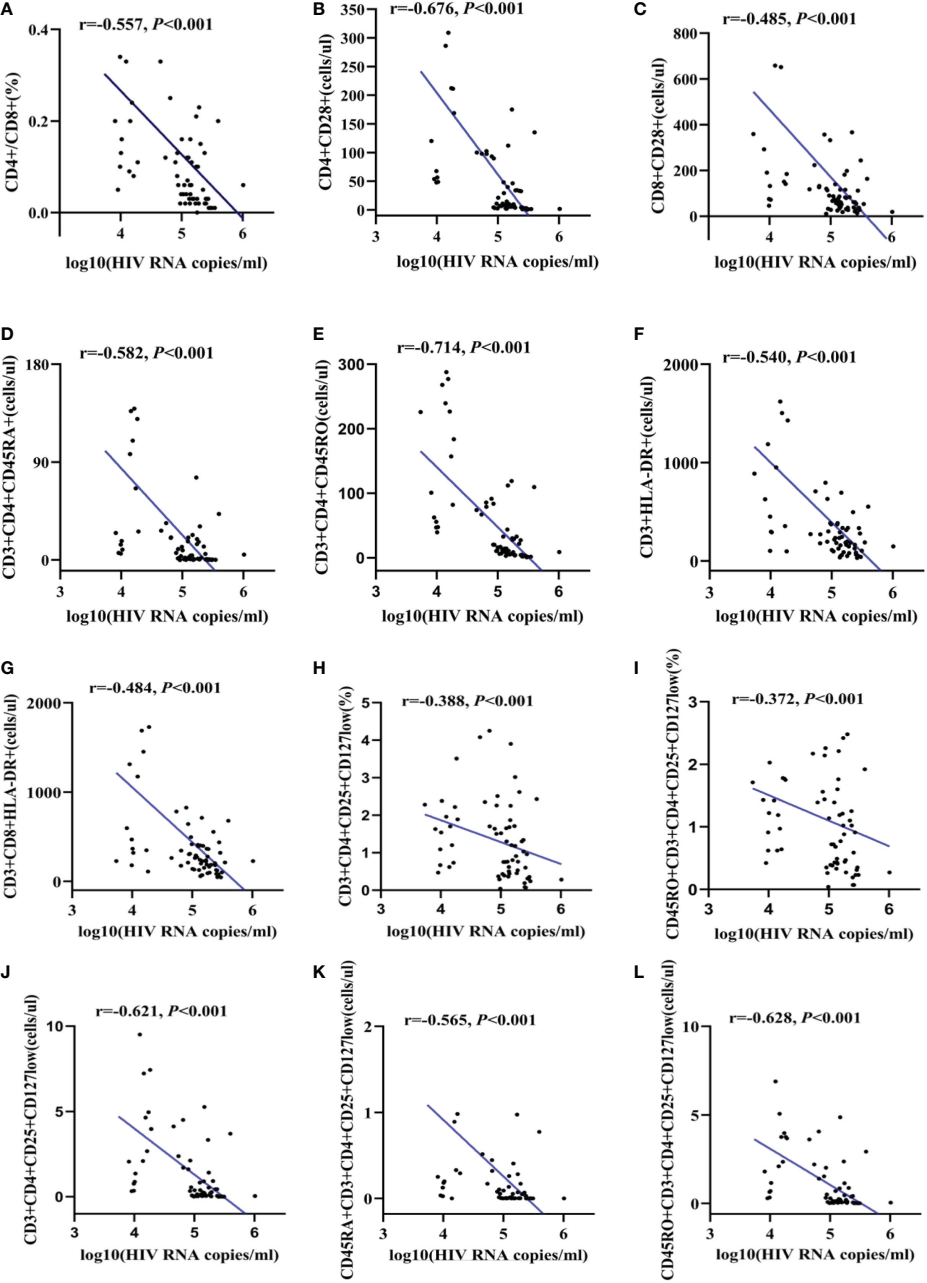
The correlation between HIV viral load and refined lymphocytes subsets. The correlation between HIV RNA and CD4/CD8 ratio **(A)**. The correlation between HIV RNA and CD28 expression **(B, C)**. The correlation between HIV RNA and naïve/memory T lymphocyte subsets **(D, E)**. The correlation between HIV RNA and activate T cell subsets **(F, G)**. The correlation between HIV RNA and regulatory T cells **(H-L)**.

According to the results in **Figure 4**, all these followed refined lymphocytes subsets were negatively correlated with HIV RNA viral load. The correlation indexes were -0.557 in CD4/CD8 ratio (**Figure 4A**), -0.676 and -0.485 in number of CD4+CD28+ T lymphocytes and CD8+CD28+ T lymphocytes, respectively (**Figures 4B, C**)). Similarly, naïve/memory T lymphocytes was significantly negatively correlated with HIV RNA viral load, with a relatively high correlation indexes in number of CD3+CD4+CD45RO T lymphocytes (r=-0.704, *p*<0.001). Besides, the correlation indexes were -0.540 and -0.484 in number of CD3+HLA-DR and CD3+CD8+HLA-DR lymphocytes (**Figures 4F, G**)). As for regulatory T cells, with the exception of a relatively low correlation indexes in the ratios of CD3+CD4+CD25+CD127low cells and CD45RO+CD3+CD4+CD25+CD127low cells (r<0.5) (**Figures 4H, I**), the correlation indexes in number of CD3+CD4+CD25+CD127low, CD45RA+CD3+CD4+CD25+CD127low cells and CD45RO+CD3+CD4+CD25+CD127low cells were -0.621, -0.565 and -0.628, respectively (**Figures 4J-L**).

## Discussion

It has been shown that immune system, including various types of immune cells and cytokines, will be obviously affected as the progression of disease after HIV infection, with a decrease in the proportion of CD4+ T lymphocytes and an increase in CD8+ T lymphocytes. Additionally, there is also a regular variation of other T lymphocyte subsets, NK cells and B cells following disease progression. Fortunately, ART is known to significantly reduce HIV RNA levels in plasma and lymphoid tissue (5) and can improve immune abnormalities (6–8). On the basis of the current routine monitoring of CD4+ T lymphocytes, it is particularly necessary to further comprehensively monitor and evaluate the changes of absolute number, proportion and function of lymphocytes, and the degree of abnormal immune activation status during ART, through the monitoring of a variety of lymphocytes expressing different molecular markers and with different biological functions.

It was found that HIV infection could weaken the immune system, exacerbating immunodeficiency in a setting of immune activation in which CD4+ T lymphocytes depletion might be crucial (9), while CD8+ T-cells are abnormally activated and increased in number. The detection of T-lymphocyte subsets, especially CD4+ T-lymphocyte counts, is an essential indicator for determining the progression of disease in patients during ART (10), formulating clinical drug protocols (11), and evaluating therapeutic and prognostic effects (12). Another characteristic of deficiency in cytoimmune function is that the ratio of CD4/CD8 reduced or even inverted (13). Thereby, the current available lymphocyte test, which includeds CD4+, CD8+ T lymphocytes and CD4/CD8 ratio, has became the routine surveillance item to estimate the reconstruction of cytological immune function in PLWHA during ART period (14). In this study, with the increase of duration time of ART, it showed a gradually increasing tendency of both CD4+ T lymphocytes and CD4/CD8 ratio, and an irregular changes of CD8+ T lymphocytes, which is consistent with the relevant literature (15). Besides, further analysis in this study showed that for those PLWHA with ART more than 10 years, CD4+T lymphocytes could return to normal levels, though the ratio of CD4+/CD8+ may take longer, implying that long-term ART duration was favorable for CD4+T lymphocytes to achieve complete immune reconstruction. Taisheng Li et al. (16)revealed that CD4 maintained stable with aging in healthy individuals, whereas CD8 decreases yearly, which led to the increase of CD4+/CD8+ with aging. Given that ART is lifelong for PLWHA, it would be more valuable to monitor CD4+/CD8+ variation on the premise that CD4 is steadily increasing.

It is currently recognized that CD28 is the most important type of second signaling receptor for T cells and can play a co-stimulatory role in T cell activation (17). Vingerhoets et al. (18) reported a significant downregulation of CD28 expression on CD4+ and CD8+ T lymphocytes in PLWHA. In addition to that, Landay et al. (19) revealed that CD28 significantly correlated with HIV viral load, and several other studies (20, 21)declared that effective ART could induced abnormal recovery of CD28+/CD8+ T lymphocyte subsets, which further suggests that CD28 detection could be an effective indicator for evaluating disease progression in PLWHA. Therefore, the levels of CD4+CD28+ and CD8+CD28+ lymphocytes is closely associated with the duration and efficacy of antiviral therapy, and can be used as one of the important indicators for prognostic evaluation. Futhermore, a study focusing on the changes of Th/Ts second signaling receptors in healthy individuals showed that, both the absolute count and percentage of CD4+CD28+ and CD8+CD28+ lymphocytes decreased yearly with aging (16). In this study, it was discovered that the number of CD4+CD28+ as well as the counts and ratio of CD8+CD28+ lymphocytes increased with ART duration time, almost reaching the levels of healthy individuals after ART initiation for more than ten years, which confirmed again that continuous ART has a significant effect on the elevation of second signaling receptor for T cells. Thus, combined with the increasing age of PLWHA and the effects of HIV itself on Th/Ts second signaling receptors, we believed that the dynamic monitoring of CD4+CD28+ and CD8+CD28+ lymphocytes for a single individual who needs lifelong ART, could provide a more objective assessment of immune reconstruction.

CD45RA and CD45RO are two allozymes of the CD45 molecule (a common leukocyte antigen). Expression of RA and RO represents two functionally distinct subsets of T lymphocytes:T cells expressing CD45RA, also known as naive T lymphocytes, being cells that have just been transferred from the thymus to the peripheral circulation without having been exposed to antigens, which have a suppressive induction effect. The other are CD45RO cells, also called memory T cells, which have a paracrine role in the induction of immunoglobulin synthesis. It was shown that HIV infection in humans results in significant changes in peripheral blood T lymphocyte subsets, as evidenced by a decrease in the number of naïve T lymphocytes and an increase in the number of memory T lymphocytes, with a continuous decrease in CD45RA directly correlating with the sustained production of D45RO cells (22, 23). After ART was initiated, memory CD4+ T lymphocytes and naïve CD4+ T lymphocytes increased to varying degrees, in accordance with the results observed in this study. Moreover, Ullum H et al. suggested that the number of CD4+CD45RA+ cells predicted AIDS progression and death (24), therefore, continuous monitoring of naive/memory lymphocyte subsets in PLWHA is of great importance to predict disease progression and ART efficacy (25). Considering the effect of age on the disease, relevant literature was reviewed and analyzed, with a study by Taisheng Li et al. showing (16)that with age, the absolute number and percentage of naive CD4+ T cells decreased annually in healthy individuals; the absolute number and percentage of memory CD4+ T cells increased yearly. Furthermore, our findings showed that both naive CD4+ T cells and memory CD4+ T cells increased with prolonged treatment, the ratio of naive CD4+ T cells increased while the ratio of memory CD4+ T cells decreased. In this study, a exciting result showed that all these indexes could reach the levels of healthy people after ten years of ART. Consequently, combining the effects of age and HIV as well as ART treatment on naive/memory cells and our findings, we tentatively suggest that a rise in both naive CD4+ T cells absolute counts and percentages better reflects the restoration of immune reserve capacity in patients with AIDS, excluding age interference.

HLA-DR are MHC-II molecules instead of markers of viral particle producing cells, where they are present on cells with a high proportion of intact viral sequences and high proliferative potential, whose overexpression on T cells can reflect the overactivation or immaturity of immune cells (26). It was found that HIV infection leads to a significant increase in the proportion of HLA-DR molecules expressed on T lymphocytes and a decrease in HLA-DR expression after ART treatment (18, 26, 27).Phillips A N et al. showed that the level of CD8 T cell activation expressed by HLA-DR was better associated with risk of AIDS morbidity and mortality (28). Moreover, Horsburgh B A et al. emphasized the importance of HLA-DR+ cells in carrying 56% of genetically intact and potentially replication-competent viruses during effective antiretroviral therapy (29). Taken together, sustained immune activation is a hallmark of HIV infection in humans as a key driver of disease progression. HLA-DR is of great significance in monitoring HIV disease progression and clinical efficacy. Similarly, our results also revealed that the counts and percentages of activated lymphocytes declined gradually after ART, although they were unable to return to normal levels even after the intiation of ART for ten years. This suggests that ART can inhibit the organism’s abnormal activation state after HIV infection, yet it may take much longer time to achieve immune reconstitution, and the exact mechanism need to be further investigated. Besides, the Li Taisheng et al. (16) showed that the percentage of activated Ts cells increased annually with age in healthy individuals. In our study, the longer the duration of treatment, the lower the percentage of activated T cells and Ts cells with a tendency of regularity. In a comprehensive analysis of the specific effects of age on lymphocytes we propose that it is more comparable to apply the fraction of activated T cells and Ts cells to measure the aberrant immune activation status of PLWHA.

Tregs are a group of cells with immunomodulatory functions that play an important role in maintaining autoantigen tolerance, limiting chronic inflammation and regulating the homeostatic balance of lymphocyte proliferation,which have been revealed to consist mostly of two types of phenotypically and functionally distinct subsets: natural regulatory and induced regulatory T cells (30). It was found that Tregs are involved in the immunoregulation process after HIV infection (31), although the roles of regulatory function of immune cells in PLWHA were controversial (32). Some studies considered Tregs played a detrimental role as they could inhibit antiviral T-cell responses, whilst others thought Tregs played a beneficial role through the reduction of immune activation (33, 34). Moreover, Tregs have been shown to control the activation status of HIV-infected lymphocytes because increased immune activation is a hallmark after HIV infection (35). Another study found that the T lymphocyte counts and percentages of those ART-initiated PLWHA were significantly higher than those ART-naive PLWHA, which is consistent with the results of this study. In our study, we noticed that the counts of regulatory T cells increased significantly with the extension of ART treatment duration and gradually rised to a relative high level after 10 years of ART, including both CD45RA+CD3+CD4+CD25+CD127low and CD45RO+CD3+CD4+CD25+CD127low T lymphocytes.These data indicated that continuous ART was helpful to increase the number and proportion of regulatory T lymphocyte subsets. Taken together, these studies suggested that Tregs may play a immunoregulation role in slowing down disease progression after HIV infection. Therefore, we Conclude that as the immune function of HIV-infected patients is re-established after ART, the number of regulatory Th cells counts also gradually increases, which has an important regulatory role in alleviating the aberrant immune activation status of patients.

Overall, there were some highlights in this study. Firstly, characteristics of changes in functional lymphocytes subsets, naive/memory lymphocyte subsets, activated lymphocyte subsets as well as regulatory lymphocyte subsets during ART period, instead of merely conventional CD4+ T lymphocytes detection, were comprehensively analyzed. Then, dynamic changes of lymphocyte subsets with different time of ART implementation were evaluated, and some PLWHA received ART even longer than 10 years. Furthermore, the levels of lymphocyte subsets in PLWHA received ART longer than 10 years were compared with healthy individuals, which may provide preliminary clinical evidence for predicting the time required for complete immune restoration in PLWHA.

However,we recognize that our study has some limitations. Firstly, most of PLWHA were males, which might inevitably leads to a certain degree of selection bias. However, these data objectively reflected the current situation that more males than females had been infected with HIV in China. In order to further eliminate the influence of age, gender and other confounding factors on refined lymphocyte subsets changes during the duration of ART, future study would be conducted to enlarge the sample size. Secondly, the influence of baseline lymphocyte level, clinically relevant underlying diseases and AIDS-related opportunistic infections were not analyzed in this study, which needs further improvement. Thirdly, this is a cross-sectional study, and a cohort for systematic monitoring would more accurately demonstrate the dynamic patterns of various lymphocyte subsets. Even so, this cross-sectional study could provide clinical evidence for the importance of refined lymphocyte subsets monitoring. Lastly, the number and functions of NK cells can be impaired in HIV-infected patients with high viral loads. CD56dimCD16bright mature NK cells are often dramatically expanded and hyper responsive in human immunodeficiency virus infection. CD56brightCD16dim immature NK cells can play a role of adaptive response and regulatory NK cells in early immune responses. Further refined NK cells monitoring can better distinguish the effect of ART on NK cells of different subsets, which was not carried out in this study and would be focused on this subsets testing in the future.

In summary, ART could improve the immune status of PLWHA, and the duration time of ART could determine the degrees of immune reconstruction. The dynamic monitoring of refined lymphocyte subsets in PLWHA, could make a more comprehensive assessment of lymphocyte numbers, lymphocyte function and abnormal activation status of the immune system, so as to make a more accurate analysis on the efficacy of ART and prognosis.

## Data availability statement

The original contributions presented in the study are included in the article/supplementary material. Further inquiries can be directed to the corresponding author.

## Ethics statement

The studies involving human participants were reviewed and approved by Ethics Committee of Zhongnan Hospital of Wuhan University. The patients/participants provided their written informed consent to participate in this study.

## Author contributions

RY and RRY conceived of and designed the study. LL, LF, XC and QC collected samples. WH and YY performed the experiments. EZ and KZ analyzed the data. HK and XG provided suggestions. All authors contributed to the study and approved the submitted version.
